# Pharmacodynamics of the Novel Antifungal Agent F901318 for Acute Sinopulmonary Aspergillosis Caused by *Aspergillus flavus*

**DOI:** 10.1093/infdis/jix479

**Published:** 2017-09-12

**Authors:** Clara E Negri, Adam Johnson, Laura McEntee, Helen Box, Sarah Whalley, Julie A Schwartz, V Ramos-Martín, Joanne Livermore, Ruwanthi Kolamunnage-Dona, Arnaldo L Colombo, William W Hope

**Affiliations:** 1 Laboratório Especial de Micologia, Disciplina de Infectologia, Escola Paulista de Medicina, Universidade Federal de São Paulo, Brazil; 2 Antimicrobial Pharmacodynamics and Therapeutics, University of Liverpool, United Kingdom; 3 Charles River Laboratories, Davis, California; 4 Department of Biostatistics, Institute of Translational Medicine, University of Liverpool, United Kingdom

**Keywords:** *Aspergillus flavus*, in vivo, orotomides, pharmacodynamics, pharmacokinetics

## Abstract

**Background:**

*Aspergillus flavus* is one of the most common agents of invasive aspergillosis and is associated with high mortality. The orotomides are a new class of antifungal agents with a novel mechanism of action. An understanding of the pharmacodynamics (PD) of the lead compound F901318 is required to plan safe and effective regimens for clinical use.

**Methods:**

The pharmacokinetics (PK) and PD of F901318 were evaluated by developing new in vitro and in vivo models of invasive fungal sinusitis. Galactomannan was used as a pharmacodynamic endpoint in all models. Mathematical PK-PD models were used to describe dose-exposure-response relationships.

**Results:**

F901318 minimum inhibitory concentrations (MICs) ranged from 0.015 to 0.06 mg/L. F901318 induced a concentration-dependent decline in galactomannan. In the in vitro model, a minimum concentration:MIC of 10 resulted in suppression of galactomannan; however, values of approximately 10 and 9–19 when assessed by survival of mice or the decline in galactomannan, respectively, were equivalent or exceeded the effect induced by posaconazole. There was histological clearance of lung tissue that was consistent with the effects of F901318 on galactomannan.

**Conclusions:**

F901318 is a potential new agent for the treatment of invasive infections caused by *A flavus* with PDs that are comparable with other first-line triazole agents.

## Teaser

F901318 is an orotomide, which is a new class of antifungal agent with activity against *Aspergillus* spp. Here, we describe the pharmacodynamics of F901318 against *Aspergillus flavus* and define the drug exposure targets required for efficacy in clinical studies.

*Aspergillus flavus* is one of the most common causes of both invasive and noninvasive fungal rhinosinusitis [1–5]. Acute invasive fungal rhinosinusitis is a relatively uncommon, but rapidly progressive lethal disease with mortality rates of 20%–80% [6–9]. Treatment requires early aggressive antifungal therapy. Surgical debridement may also be required [5, 6, 8]. Voriconazole or a lipid formulation of amphotericin B are first-line antifungal agents for treatment of acute invasive sinusitis [10]. Triazole resistance is being increasingly reported in clinical strains of *A flavus* [11–16]. As a result, new antifungal agents are urgently required.

F901318 (F2G Ltd., Eccles, UK) is the lead compound of the orotomides, which is a new antifungal class. F901318 reversibly inhibits dihydroorotate dehydrogenase and interferes with pyrimidine biosynthesis. Potent in vitro and in vivo activity has been demonstrated against a wide spectrum of pathogenic filamentous fungi including *Penicillium* spp, *Coccidiodes immitis*, *Histoplasma capsulatum*, *Blastomyces dermatitidis*, *Fusarium* spp, *Scedosporium* spp, and *Aspergillus* spp such as *Aspergillus fumigatus* and *A flavus* [17, 18].

In this study, we describe the pharmacodynamics (PD) of F901318 against *A flavus*. We developed new in vitro models of acute invasive sinusitis that enabled the PD of voriconazole and F901318 to be elucidated. A murine model of sinopulmonary invasive aspergillosis resulting from *A flavus* was also developed and used to evaluate the PD of F901318. The results were benchmarked against the PD of posaconazole.

## METHODS

### *Aspergillus flavus* Strains and Antifungal Susceptibility Testing

Four *A flavus* isolates were used in this study (Table 1). ATCC204304 was used for the primary development of new in vitro models of invasive *A flavus* infection. An additional 3 clinical *A flavus* isolates that had caused invasive disease were selected from the Special Mycology Laboratory (LEMI) (UNIFESP, Brazil). LEMI764 was isolated from a skin biopsy, LEMI1024 was isolated from a hepatic lesion, and LEMI1049 was isolated from nasal mucosa. Strains were identified as *A flavus* sensu stricto based on standard morphological and molecular studies, including sequence analysis of the internal transcribed spacer region, calmodulin, and β-tubulin genes [11, 19].

Minimum inhibitory concentration (MIC) values were estimated according to European Committee on Antimicrobial Susceptibility Testing and Clinical Laboratory Standards Institute methodologies [20, 21]. Compound with >98% purity was used for susceptibility testing. All susceptibility tests were performed as 10 independently conducted experiments for posaconazole (Sigma-Aldrich, Dorset, UK), voriconazole (Sigma-Aldrich, Dorset, UK), and F901318 (F2G Ltd., Manchester, UK).

### Development of a Cellular Bilayer Model of Human Nasal Epithelium

A previously described in vitro model of the human alveolus was modified to simulate acute invasive sinusitis caused by *A flavus* [22, 23]. A cellular bilayer with human nasal epithelial cells ([HNECs] Promocell, Heidelberg, Germany) and human pulmonary artery endothelial cells ([HPAECs] Promocell) was constructed. Human nasal epithelial cells were cultured into ready-to-use airway epithelial cell growth medium (Promocell). The HPAECs were cultured in endothelial growth medium ([EGM]-2), prepared according to the manufacturer’s instructions (amphotericin B and gentamicin were not added). Cells were incubated at 37°C in 5% CO_2_. Once confluence was achieved, cells were washed twice with Hanks Balanced Salt Solution (Sigma-Aldrich) and harvested with warmed 0.25% trypsin-ethylenediaminetetraacetic solution. Cells were centrifuged for 5-minutes and resuspended in warmed media to achieve final concentrations of 1 × 10^6^ cells/mL for both HNECs and HPAECs.

Cellular bilayers were constructed on ThinCert cell culture inserts. Different sizes of inserts were used for the static and dynamic models. Inserts were inverted onto glass sterile petri dishes, and HPAECs cells were placed on the underside of the membrane. To establish adherence, the inverted inserts were incubated for 2 hours at 37°C in 5% CO_2_. Inserts were then righted and transferred to plates containing EGM-2 medium. After 24 hours, inserts were relocated to new plates with fresh EGM-2 medium and HNECs were seeded on the top of the membrane. The medium from the endothelial compartment was changed every 48 hours for 5 days. Accumulated medium from epithelial compartment was removed. The integrity of the bilayer was assessed using the translocation of 1% Blue Dextran (Sigma-Aldrich) [22].

At the time of *A flavus* infection, inserts were placed into plates containing EBM-2 media supplemented with 2% fetal bovine serum (FBS) (Lonza Biologics, Basel, Switzerland). A suspension containing 1 × 10^4^ conidia/mL was pipetted into the nasal epithelial compartment (100 µL and 400 µL for static and dynamic experiments, respectively). Inserts were incubated for 6 hours at 37°C in 5% CO_2_, after which the inoculum was removed and discarded.

To characterize the time-course of invasion, inserts were fixed in Cellstor pot—90 mL with 10% neutral-buffered formalin (CellPath, Newtown, UK) for histopathological studies. Sections were stained with Grocott’s Methenamine Silver stain using standard protocols.

### Development of a Dynamic In Vitro Model of Acute Invasive Sinusitis

Custom-designed and previously described [22] stainless steel bioreactors were incorporated in circuits constructed with Marprene thermoplastic elastomer tubing (Watson Marlow, Falmouth, UK), Silastic, 1.6-mm bore tubing (Dow Corning, Barry, UK), and polypropylene barbed luer adapters (West Group, Waterlooville, UK). A 250-mL Duran bottle was used as a central compartment, and contained 200-mL warmed Dulbecco’s modified Eagle medium (DMEM) containing d-glucose, l-glutamine, and HEPES buffer (Invitrogen, Paisley, UK) supplemented with 2% FBS and penicillin-streptomycin solution (Sigma). The central compartment was connected into the circuit with 1.5-mm bore polytetrafluoroethylene semirigid tubing (Kinesis, St Neots, UK) fitted into Omni-Fit Q-series bottle caps (Kinesis). To ensure continuous mixing, magnetic stirring bars were used. An additional 2 Duran bottles were used: one containing fresh supplemented DMEM medium, and one empty to collect spent waste medium.

All circuits were primed with medium before use. The pump between central compartments and bioreactors was set to produce a flow rate of approximately 10 mL/hour. The pump that connected waste and fresh medium to the central compartment for voriconazole and F901318 ran at ~10 and 23 mL/hour, respectively. In both cases, the goal was to simulate human-like pharmacokinetics (PK).

### Pharmacodynamic Experiments With Voriconazole and F901318

For PD studies in the static model, voriconazole and F901318 were diluted in EBM-2 media supplemented with 2% FBS. A final volume of 600 µL per well was used in 7 different concentrations that ranged from 0.03 to 2 mg/L and 0.015 to 1 mg/L for voriconazole and F901318, respectively. Infected inserts were exposed to drug-containing plates for 48 hours. Subsequently, medium from the endothelial compartment as well as an epithelial lavage with 300 µL phosphate-buffered saline was collected for galactomannan estimation.

For the PD studies in the in dynamic model, target concentrations of 0.6, 0.25, 1, and 2 mg/L voriconazole were studied in addition to a drug-free control circuit. Voriconazole was administered at 6, 18, 30, 42, 54, and 66 hours postinoculation. Pharmacokinetic and PD samples were collected from taps inserted in each circuit between 6 and 78 hours postinoculation.

F901318 was administered as a continuous 4-hour infusion prepared using a β-hydroxypropyl cyclodextrin vehicle [24]. F901318 infusions were prepared to achieve final concentrations of 0.015, 0.06, 0.25, and 1 mg/L. The PK-PD samples were taken between 6 and 78 hours postinoculation for all circuits.

### Murine Model of Acute Sinopulmonary Aspergillosis

A new neutropenic murine model of sinopulmonary invasive aspergillosis caused by *A flavus* was developed by using galactomannan as the primary PD readout. Experimentation was performed under UK Home Office project license PPL40/3630 and had received approval from the Animal Welfare Ethics Review Board of The University of Liverpool. Male CD1 mice were purchased from Charles River and weighed 25–30 grams at the time of experimentation. Food and water were provided ad libitum. Mice were rendered neutropenic with intraperitoneal injection of cyclophosphamide 150 mg/kg on day −4 and 100 mg/kg on day −1 relative to infection. Mice were further immunosuppressed with cortisone acetate (250 mg/kg) subcutaneously on day −1 to impair pulmonary macrophage function. Drinking water was supplemented with 2.5% enrofloxacin to prevent opportunistic bacterial infections.

For inoculation, mice were anesthetized with 2% isofluorane. A suspension of conidia containing 5 × 10^5^ colony-forming units (CFU)/mL (50 µL/mouse of 1 × 10^7^ CFU/mL) was instilled in both nares. The administration of antifungal compounds was delayed for 6 hours postinoculation. At predefined sampling times, groups of mice (n = 3) were terminally anesthetised using 5% isofluorane, and blood was obtained via cardiac puncture. The lungs and nasal cavities of each of the mice were removed and placed in 10% neutral-buffered formalin for histopathological analyses. Galactomannan (GM) serum indices were measured using a commercial kit (Platelia *Aspergillus* Kit; Bio-Rad).

### In Vivo Pharmacokinetics and Pharmacodynamics of F901318

Multiple independently conducted experiments were performed to determine the PK-PD of F901318 against 4 strains of *A flavus*. F901318 regimens of 24 mg/kg per day, 8 mg/kg q8h, and 15 mg/kg q8h, and a vehicle control were used. These regimens were chosen based on previous studies in *A fumigatus* [24]. A positive control cohort of mice that received posaconazole 20 mg/kg per day orally was included. Such a posaconazole regimen results in drug exposure that is significantly more than that achievable in the clinic and ordinarily results in complete suppression of the GM index. Mice were anaesthetized with 5% isofluorane, and blood was obtained via cardiac puncture. The plasma and/or serum was removed and stored at −80°C until future analysis. The plasma PK of F901318 was determined using liquid chromatography-tandem mass spectrometry (LC/MS/MS). For the PD, GM levels in serum were measured as described above.

Although voriconazole is a first-line agent for the treatment of all forms of invasive aspergillosis, it is notoriously difficult to study in mice because of its short half-life and autoinduction of clearance [25]. In contrast, posaconazole has much more predictable behavior with established in vivo-to-clinical relationships [26]. For this reason, the PD of F901318 were benchmarked against posaconazole, which is consistent with the recent approach taken by us [27]. The PK-PD relationships of posaconazole against *A flavus* were determined by studying the effect of 2.5, 5, 10, and 20 mg/kg per day. An area under the curve (AUC) of 47 mg*h/L is the upper 95% confidence bound of the upper quartile of response in patients with invasive aspergillosis receiving posaconazole as salvage therapy [28].

### Survival Experiments

F901318 was administered as 24 mg q24h, 8 mg/kg q8h, and 15 mg/kg q8h instravenously (i.v.) for 3 days. These regimens were based on previous studies against *A fumigatus* [24]. Posaconazole was administered as 2.5, 5, 10, and 20 mg/kg per day. Each cohort consisted of n = 10 mice. After treatment ceased, mice were observed for an additional 7 days. Mice were sacrificed if they were terminally unwell, could not move to food and water, or had lost >20% of their original body weight.

### Statistical Modeling

Cox proportional hazard models were fitted to the survival data from mice infected with each A flavus strain and receiving F901318, and mice infected with LEMI764 and receiving posaconazole. The minimum blood plasma concentration (*C*_min_):minimum inhibitory concentration (MIC) and AUC were used as covariates to account for differences in survival in each of the treatment cohorts receiving different regimens F901318 and posaconazole, respectively. The model took the form $\text{λ}\left(\text{t}\right)\;={\text{ λ}}_{0}\left(\text{t}\right)\exp(\text{γX})$, where λ(t) is the hazard, ${\text{λ}}_{0}\left(\text{t}\right)$ is the baseline hazard, X is the covariate. The function exp(γ) provides an estimate of the hazard ratio.

### Bioanalysis via Liquid Chromatography-Tandem Mass Spectrometry

Antifungal drug concentrations from in vitro and in vivo samples were measured using LC/MS/MS with an Agilent 6420 Triple Quad Mass spectrometer and Agilent 1290 infinity LC system (Agilent Technologies UK Ltd, Cheshire, UK). Drug concentrations were measured in cell culture medium (voriconazole and F901318) and mouse plasma (posaconazole and F901318). The assays are described in detail in Supplementary Data.

### Pharmacokinetics and Pharmacodynamic Mathematical Modeling

The PK-PD mathematical model was fitted to the data using the population PK program Pmetrics [29]. In these analyses, an “individual” consisted of a cohort of mice receiving a given regimen of F901318 or posaconazole.

The combined PK-PD datasets (ie, plasma concentrations of F901318 and posaconazole and corresponding galactomannan data) were comodeled. The structural model shown below is for F901318. The same model was used for posaconazole except that the absorptive compartment was used to account for the oral administration of drug.

$$XP\left(1\right)=\;R\left(1\right)-\left(\frac{SCL}{V}\right)\text{*}X\;[1]-Kcp\cdot X\left(1\right)+Kpc\cdot X\;[2]$$ 1

XP(2)= Kcp⋅X(1)−Kpc⋅X [2] 2

$$\text{XP}\left(3\right)=\text{ Kgmax}\cdot \left(1-\left(\frac{{\left(\frac{\text{X}\left(1\right)}{\text{V}}\right)}^{\text{Hg}}}{\text{C}50{\text{g}}^{\text{Hg}}+{\left(\frac{\text{X}\left(1\right)}{\text{V}}\right)}^{\text{Hg}}}\right)\right)\cdot \text{X}\left(3\right)$$ 3

$$\text{*}\left(1-\left(\frac{\text{X}\left(3\right)}{\text{popmax}}\right)\right)-\text{kkillmax*}\left(\frac{{\left(\frac{\text{X}\left(1\right)}{\text{V}}\right)}^{\text{Hk}}}{\text{C}50{\text{s}}^{\text{Hk}}+{\left(\frac{\text{X}\left(1\right)}{\text{V}}\right)}^{\text{Hk}}}\right)\text{*}\cdot \text{X}\left(3\right)$$

With output equations

Y [1]=X [1]/V

Y [2]=X [3]

The system parameters and their units are as follows: R [1] represents the i.v. injection of F901318. SCL (L/h) is the clearance of drug from the central compartment; V (L) is the volume of the central compartment; Kcp (h^−1^) and Kpc (h^−1^) are the first-order intercompartmental rate constants. Kgmax (GM/h) and kkillmax (GM/h) are the maximal rates of fungal growth and drug-induced kill, respectively. Popmax (GM) is the maximum theoretical fungal density. C50g (mg/L) and C50k (mg/L) are the concentrations of F901318 (or posaconazole) that induce half-maximal effects on growth and kill, respectively. Hg and Hk are the respective slope functions for growth and kill. The initial condition (GM; data not shown in the equations) is the fungal density immediately after inoculation, and it is estimated along with other parameters.

Equations 1 and 2 are standard PK equations that describe a 2-compartment PK model with first-order clearance from the central compartment. The PD of F901318 (or posaconazole) against *A flavus* is described by Equation 3. The first and second output equations provides the time course of F901318 plasma concentrations and galactomannan, respectively.

## RESULTS

### *Aspergillus flavus* Strains and Antifungal Susceptibility Tests

The estimates for the MICs of F901318, voriconazole, and posaconazole for the 4 *A flavus* isolates used in this study are summarized in Table 1.

**Table 1.** *Aspergillus flavus* Isolates Used in this Study^a^

Strain	Antifungal Agent	CLSI			EUCAST		
		Range	Mode	G. Mean	Range	Mode	G. Mean
ATCC204304	Voriconazole	0.5–1	0.5	0.6	1–2	1	1.3
	Posaconazole	0.06–0.125	0.125	0.09	0.06–0.25	0.125	0.12
	F901318	0.008–0.03	0.008	0.011	0.03–0.06	0.03	0.03
LEMI764	Voriconazole	0.25–1	0.25	0.3	1–2	1	1.1
	Posaconazole	0.03–0.125	0.06	0.06	0.125	0.125	0.125
	F901318	0.008–0.015	0.015	0.01	0.015–0.06	0.03	0.027
LEMI1024	Voriconazole	0.25–1	0.5	0.6	1–2	2	1.7
	Posaconazole	0.25	0.25	0.25	0.125	0.125	0.125
	F901318	0.008–0.03	0.008	0.012	0.03–0.06	0.03	0.03
LEMI1049	Voriconazole	0.125–1	0.5	0.3	1–2	1	1.1
	Posaconazole	0.06–0.125	0.06	0.06	0.125	0.125	0.125
	F901318	0.015–0.03	0.03	0.023	0.03–0.06	0.03	0.04

Abbreviations: CLSI, Clinical Sciences Laboratory Institute; EUCAST, European Committee on Antimicrobial Susceptibility Testing; G., geometric; MICs, minimum inhibitory concentrations.

^a^The MICs from challenge strains used in this study. The MICs were determined in 10 independently conducted experiments according to CLSI and EUCAST methodology.

### Static In Vitro Model of Acute Invasive Sinusitis

**Figure 1.**
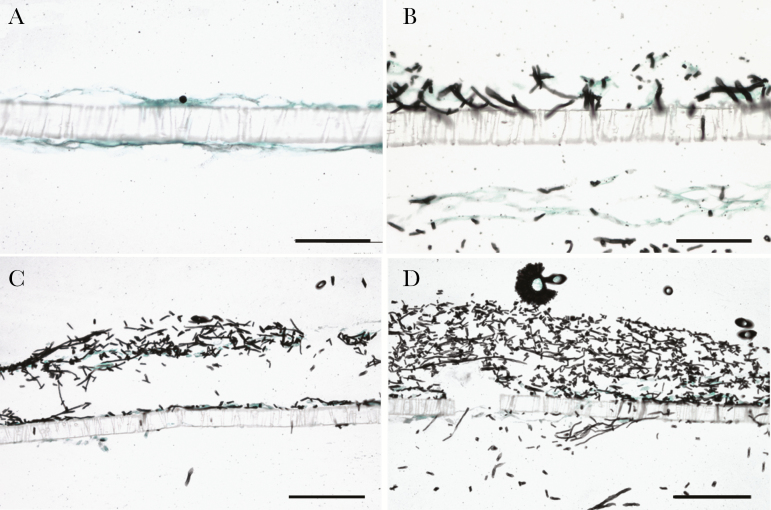
Invasion of the cellular bilayer by *Aspergillus flavus* ATCC204304. (A) At time zero, only conidia were observed in epithelial surface; (B) after 24 hours, germination began from the nasal epithelial surface with evidence of hyphal invasion through membrane pores and into the endothelium and endothelial compartment; (C) progressive fungal growth and invasion; (D) development of fruiting bodies on the epithelial surface at 72 hours postinfection. Scale bar, 25 µm (A and B) and 50 µm (C and D).

The sequence of conidial germination and hyphal invasion across the cellular bilayer is shown in Figure 1. Immediately after infection, conidia were observed on the epithelial surface (Figure 1A). Subsequently, hyphae were observed invading across the cellular bilayer and through the 3-µm perforations in the insert membrane as they transgressed from the nasal epithelial surface through to the endothelial compartment.

**Figure 2.**
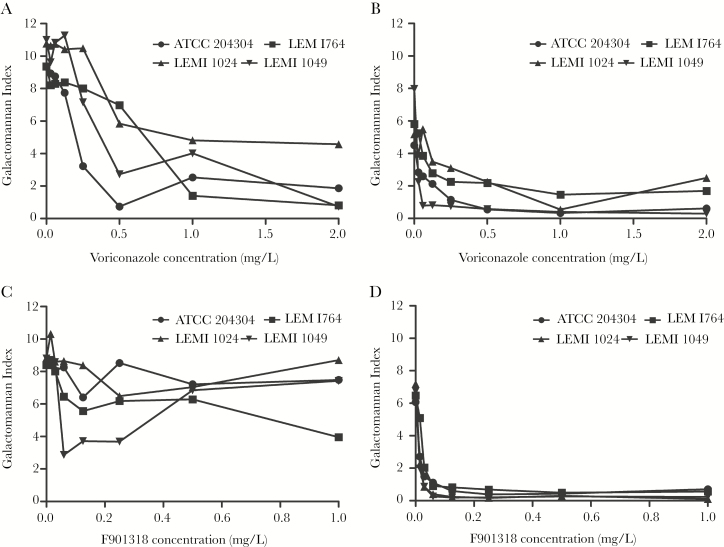
Pharmacodynamics of voriconazole and F901318 against *Aspergillus flavus*. The pharmacodynamic readout is the galactomannan index determined using the Platelia kit. (A) Galactomannan index from the nasal epithelial compartment after voriconazole exposure; (B) galactomannan index from endothelial compartment after voriconazole exposure; (C) galactomannan index from epithelial compartment after F901318 exposure; (D) galactomannan index from endothelial compartment after F901318 exposure. Data are the mean from 3 inserts. Error bars have been omitted for clarity.

Both voriconazole and F901318 caused a concentration-dependent decline in galactomannan in the in vitro model of acute invasive sinusitis. This effect was most pronounced in the endothelial compartment where both agents caused near-maximal suppression of galactomannan (Figure 2). There was more strain-to-strain variability in the alveolar compartment. Neither voriconazole nor F901318 induced complete suppression of galactomannan in this compartment.

### Pharmacokinetics and Pharmacodynamic of Voriconazole and F901318 in the In Vitro Model of Acute Invasive Sinusitis

**Figure 3.**
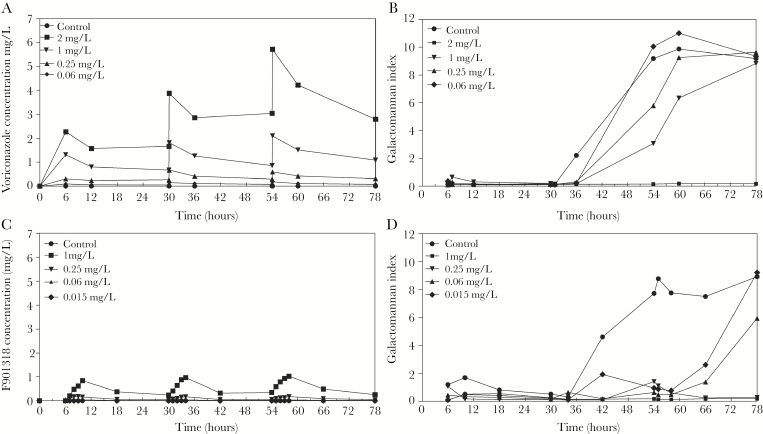
Pharmacokinetics and pharmacodynamics of voriconazole (A and B) and F901318 (C and D) in a dynamic model of acute invasive sinusitis caused by *A flavus*. The pharmacodynamic readout is the galactomannan index determined using the Platelia kit. The challenge strain was ATCC 204304. (A) Human-like pharmacokinetics of voriconazole; (B) pharmacodynamics of voriconazole; (C) human-like pharmacokinetics of F901318; (D) pharmacodynamics of F901318. A minimum blood plasma concentration (*C*_min_) of voriconazole of approximately 2 mg/L is required to suppress galactomannan. A *C*_min_ of 0.3 of F901318 (and a *C*_min_:minimum inhibitory concentration of approximately 10) is required to completely suppress galactomannan.

The simulated human PKs and the time-course of circulating galactomannan in response to voriconazole and F901318 are shown in Figure 3. Results from the ATCC strain were representative of the PD relationships of both voriconazole and F901318 against other strains of *A flavus* (data from other strains are shown in Supplementary Data). In all untreated circuits, there was a progressive rise in galactomannan after 48 hours (Figure 3). The kinetics of galactomannan in control circuits for voriconazole and F901318 were similar, suggesting that the different pump speeds did not affect the respective PD of the 2 agents. There was an exposure-dependent decline in galactomannan induced by both voriconazole and F901318. A *C*_min_ of voriconazole and F901318 of approximately 2 mg/L and 0.3 mg/L, respectively, resulted in complete suppression of circulating galactomannan.

### Histopathological Studies

**Figure 4.**
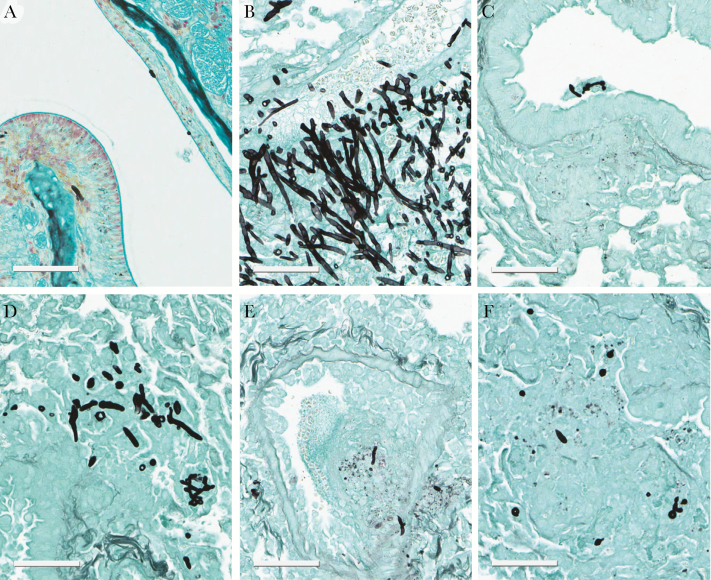
Histopathology of sinopulmonary aspergillosis. (A) *Aspergillus flavus* conidia and hyphae at and within the nasal epithelial surface 54 hours postinfection in a vehicle control; (B) section of the lung showing a fungal bronchopneumonia that was moderate-to-severe. There was evidence of vascular invasion in untreated controls 78 hours postinoculation; (C) histopathological appearances after treatment with 20 mg/kg posaconazole; (D–F) histopathological appearances in mice receiving F901318 24 mg/kg q24h, 8 mg/kg q8h, and 15 mg/kg q8h, respectively, which show a decline in fungal elements. Scale bar, 60 µm.

Relatively scant hyphal elements were evident in the nasal cavity at all time points and when present only superficial invasion was observed (Figure 4). In contrast, in the lung, there was a florid multifocal fungal bronchopneumonia that became progressively more extensive throughout the experimental period. At 78 hours postinoculation, the infection was fulminant and characterized by severe necrosis, hemorrhage, edema, necrotizing vasculitis, vascular invasion, and thrombosis. Elongating hyphae were abundant and consistently observed invading blood vessels (Figure 4B).

At 78 hours postinoculation, the appearances of F901318 24 mg/kg Q24 were significantly worse than those observed with 8 mg/kg Q8 and 15 mg/kg Q8. No fungal organisms were identified in 2 of 3 animals in the F901318 15 mg/kg Q8 dose group, and only mild infection was found in a third mouse (Figure 4F).

### In Vivo Pharmacodynamics of F901318

**Figure 5.**
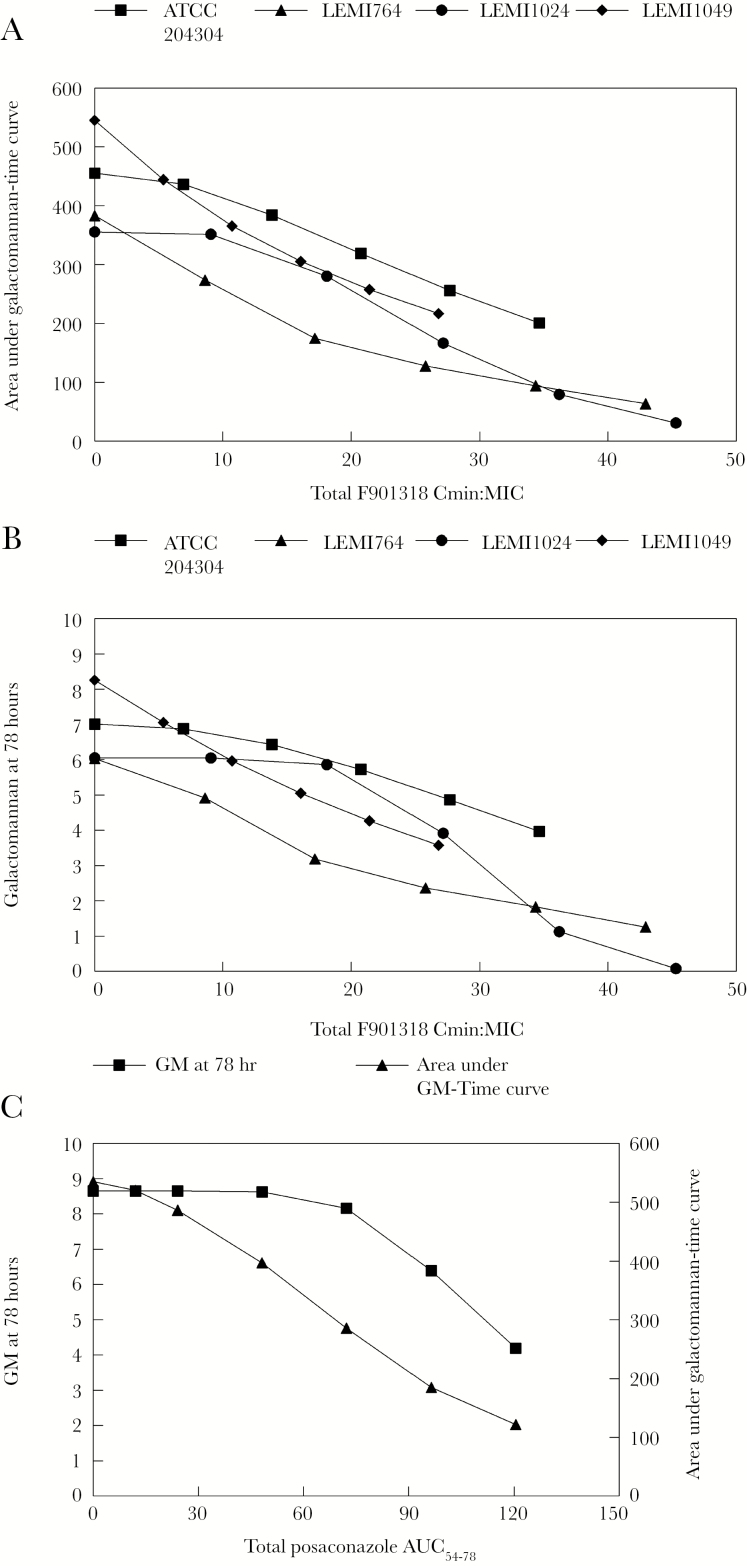
In vivo pharmacokinetic-pharmacodynamic relationships of F901318 and posaconazole against *Aspergillus flavus*. (A) Minimum blood plasma concentration:minimum inhibitory concentration (*C*_min_:MIC) versus the area under galactomannan-time curve for the 4 *A flavus* strains; (B) relationship between *C*_min_:MIC and the predicted Galactomannan (GM) (as determined by the Platelia kit) at 78 hours for 4 *A flavus* strains; (C) relationship between the posaconazole area under the curve and both the predicted GM at 78 hours (solid squares) and area under the galactomannan-time curve (solid triangles).

F901318 induced a decline in circulating galactomannan in mice with sinopulmonary aspergillosis. Near-maximal reduction was observed in mice receiving 15 mg/kg q8h for all 4 challenge strains. The use of 24 mg/kg per day was less effective than 8 mg/kg q8h, which is consistent with previous observations that F901318 exhibits time-dependent antifungal activity. The relationship between *C*_min_:MIC and GM at 78 hours as well as *C*_min_:MIC and area under GM time curve is shown in Figure 5A and B. The *C*_min_:MIC values of 9–19 (mean 13.38) induced a decline in galactomannan that matched that induced by a posaconazole AUC of 47 mg*h/L.

### Survival Studies

**Figure 6.**
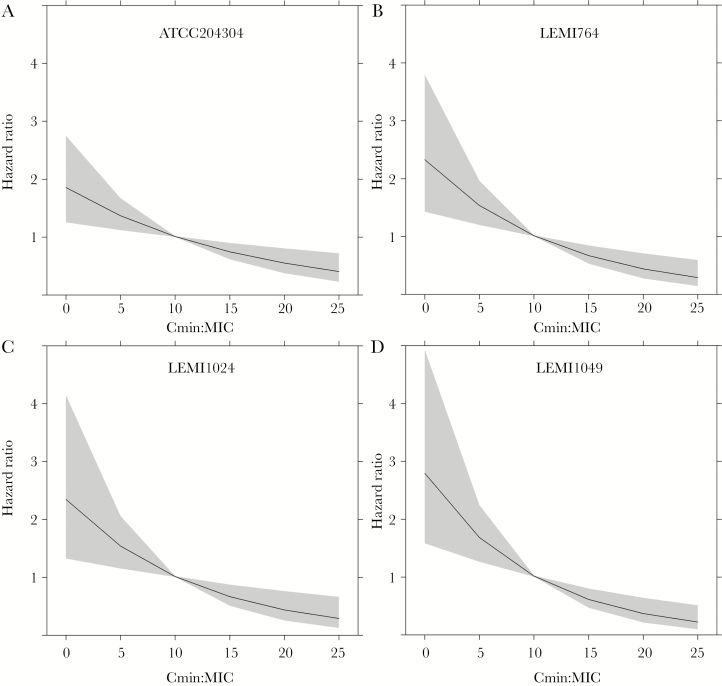
Predictions from the Cox models fitted to the survival data from mice infected with various *Aspergillus flavus* strains, treated with F901318 for 3 days, and then observed for 7 more days. The solid line is the model estimate for each strain, and the gray-shaded area represents the 95% confidence interval. The F901318 minimum blood plasma concentration:minimum inhibitory concentration (*C*_min_:MIC) that results in a hazard ratio (HR) <1 is consistently approximately 10. For comparative purposes, a posaconazole area under the curve of approximately 40 mg.h/L results in an HR of <1.

There was a drug exposure-dependent prolongation in survival for mice receiving F901318 (see Supplementary Data). For all 4 strains, *C*_min_:MIC was highly predictive of survival (HR <1 and *P* values <<.01). For F901318, each 1-unit rise in *C*_min_:MIC resulted in a ~6%–10% (95% confidence interval [CI], 15%–2%) increase in the probability of survival. For posaconazole, each 1-unit rise in AUC resulted in a ~4% (95% CI, 6%–2%) increase in the probability of survival. Plots of the HR as a function of the *C*_min_:MIC values for F901318 are shown in Figure 6. For each strain, the HR dropped below 1 (ie, caused prolongation of survival compared with controls) with *C*_min_:MIC values of approximately 10. For posaconazole, the HR was <1 after an AUC of approximately 40 mg*h/L.

## DISCUSSION

F901318 and the orotomides have the potential to address current limitations of modern systemic antifungal therapy. These new inhibitors of pyrimidine synthesis have broad-spectrum antimould activity and specific broad-spectrum anti-*Aspergillus* activity that extends to triazole-resistant strains [17, 18, 30]. F901318 is orally bioavailable, making it potentially suitable for the longer-term consolidation therapy. The single and multiple ascending dose (SAD and MAD) Phase I studies are complete as is the oral dosing program (ClinicalTrials.gov identifiers: NCT02142153, NCT02342574, NCT02394483, and NCT02737371) [18]. Phase II studies in a variety of clinical contexts are planned and will commence shortly. Invariably, patients with invasive disease caused by *A flavus* will be enrolled in these studies.

F901318 has potent in vitro activity against *A flavus* with a modal MIC of 0.03 mg/L. *Aspergillus flavus* is the second most frequent species causing invasive aspergillosis and is the leading cause of acute invasive fungal sinusitis [7, 31]. A detailed understanding of the PD of F901318 against this pathogen ensures that regimens for clinical trials are derisked as far as possible. We developed in vitro and in vivo PD models to simulate human disease caused by *A flavus*. Human nasal epithelial cells were used to model the interaction of the relatively larger conidia of *A flavus* with the upper respiratory tract. These models proved a rigorous test for voriconazole (the in vitro models) and posaconazole (the murine model). In the dynamic in vitro model, a *C*_min_ of voriconazole of 2 mg/L was required to suppress galactomannan, which is consistent with a large body of preclinical and clinical evidence [32, 33]. In the murine model, a clinically relevant exposure of posaconazole only resulted in relatively modest antifungal activity with a 25% reduction in the area under the galactomannan-time curve induced by an AUC of 47 mg*h/L.

We have recently described the PD of F901318 against *A fumigatus*, and we demonstrated that this compound exhibits time-dependent antifungal activity [27]. The *C*_min_:MIC is the PD index that best links drug exposure with the observed antifungal effect. The principal task of this PD study is to determine the magnitude of the *C*_min_:MIC that is likely to be relevant for humans with invasive disease caused by *A flavus*. The different models and endpoints provided largely concordant information on the PD of F901318 for both *A fumigatus* and *A flavus*. The dynamic in vitro model with human-like PK suggests that an *C*_min_:MIC of approximately 10 is required to suppress circulating galactomannan and provide an effect that is comparable to voriconazole. The *C*_min_:MIC values of approximately 13 and 10 result in comparable antifungal activity to posaconazole when assessed by galactomannan and survival, respectively. These drug exposures also result in near complete histopathological clearance of the lung with appearances that were comparable to posaconazole.

There are several aspects of the design of this program that deserve emphasis. The first is the importance of a positive control to benchmark the PD. We choose 2 agents for which there is a detailed understanding of the PD against *Aspergillus* spp. A *C*_min_ of voriconazole of 1–2 mg/L is required for optimal clinical outcomes, and it was required in the dynamic model of acute invasive sinusitis to achieve suppression of galactomannan. Benchmarking enables experimental findings from models that are affected by the nuances of experimental design to be placed in a clinical context. We also used more than 1 experimental model, challenge strain, and model readout to estimate the PD of F901318. Such an approach provides confidence in conclusions from preclinical models and accounts for the PD variability that is often marked.

## CONCLUSIONS

F901318 has potent in vitro activity against *A flavus* and is highly active in both in vitro and in vivo models that are faithful mimics of invasive human disease. Regimens of F901318 for Phase II and III clinical trials must be at least as effective as other licensed antifungal agents. In this regard, these PD studies provided the basis for selection of regimens for further study in patients with invasive aspergillosis caused by *A flavus*.

## Supplementary Data

Supplementary materials are available at *The Journal of Infectious Diseases* online. Consisting of data provided by the authors to benefit the reader, the posted materials are not copyedited and are the sole responsibility of the authors, so questions or comments should be addressed to the corresponding author.

## Supplementary Material

Supplementary material

## Notes

Presented in part: 55th Interscience Conference on Antimicrobial Agents and Chemotherapy, September 2015, San Diego, California.
