# Errata

**DOI:** 10.1093/infdis/jiy145

**Published:** 2018-04-20

“Pharmacodynamics of the Novel Antifungal Agent F901318 for Acute Sinopulmonary Aspergillosis Caused by *Aspergillus flavus*” by Negri et al. [J Infect Dis 2017; doi: 10.1093/infdis/jix479]. This is an Open Access article and should have contained the following copyright line: © The Author(s) [2018]. Published by Oxford University Press for the Infectious Diseases Society of America. This is an Open Access article distributed under the terms of the Creative Commons Attribution License (http://creativecommons.org/licenses/by/4.0/), which permits unrestricted reuse, distribution, and reproduction in any medium, provided the original work is properly cited.

The authors regret this error.

