# Supplementary material for: Pharmacodynamics of the Novel Antifungal Agent F901318 for Acute Sinopulmonary Aspergillosis Caused by Aspergillus flavus
Source: J Infect Dis. 2017 Sep 12;217(7):1118–27. doi: 10.1093/infdis/jix479 (PMC5909626; doi:10.1093/infdis/jix479)
Supplement: Supplementary material [file jix479_suppl_supplementary_material.docx]

**Supplementary information for:**

**Pharmacodynamics of the Novel Antifungal Agent F901318 for Acute Sinopulmonary Aspergillosis Caused by *Aspergillus flavus***

Clara E. Negri, Adam Johnson, Laura McEntee, Helen Box, Sarah Whalley, Julie A. Schwartz, Ramos-Martín V, Joanne Livermore, Ruwanthi Kolamunnage-Dona, Arnaldo L. Colombo, William W. Hope

**Supplementary methodology description**

**Bioanalytical Methods**

F901318 was extracted by protein precipitation using 300 µL of acetonitrile: methanol (50:50) containing the internal standard (IS) 6, 7-Dimethyl-2, 3-di (2-pyridyl) quinoxaline at 1 mg/L (Sigma Aldrich, Dorset, UK) was added to 100µl of matrix. A total of 300µL of acetonitrile containing the internal standard (IS) phenacetin 0.1 mg/L (Sigma Aldrich, Dorset, UK) was added to 100µl of matrix. Chromatographic separation was achieved for F901318 using a gradient with the starting conditions of 70:30 (0.2% formic acid in water as mobile phase A and 0.2% formic acid in acetonitrile as mobile phase B). The standard curve for F901318 encompassed the concentration range of 0.001-20.0 mg/L and was constructed using blank matrix.

Voriconazole was extracted by protein precipitation. Chromatographic separation for Voriconazole was achieved using a gradient with the starting conditions of 70:30 (0.1% formic acid in water as mobile phase A and 0.1% formic acid in acetonitrile as mobile phase B). The precursor ion for voriconazole was 350.0m/z, and it was 180.1 m/z for the IS. The product ion for voriconazole was 281.1 m/z and it was 110.0 m/z for the IS. The source parameters were set as 4000 V for capillary voltage, 350°C for gas temperature, and 60 lb/in^2^ for the nebulizer gas. The standard curve for voriconazole encompassed the concentration range of 0.025-20.0 mg/L and was constructed using blank matrix.


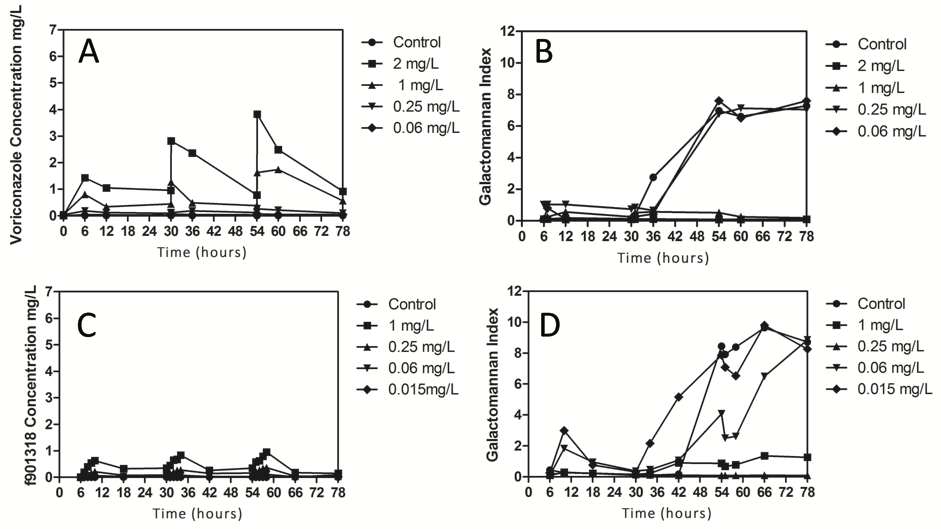


**Supplementary Figure 1.** Pharmacokinetics and pharmacodynamics of voriconazole (**A** and **B**) and F901318 (**C** and **D**) in a dynamic model of acute invasive sinusitis caused by *Aspergillus flavus* **LEMI764**. The pharmacodynamic readout is the galactomannan index determined using the Platelia kit. ***A****,* Human-like pharmacokinetics of voriconazole; ***B****,* pharmacodynamics of voriconazole; ***C****,* Human-like pharmacokinetics of F901318; ***D****,* pharmacodynamics of F901318.

**
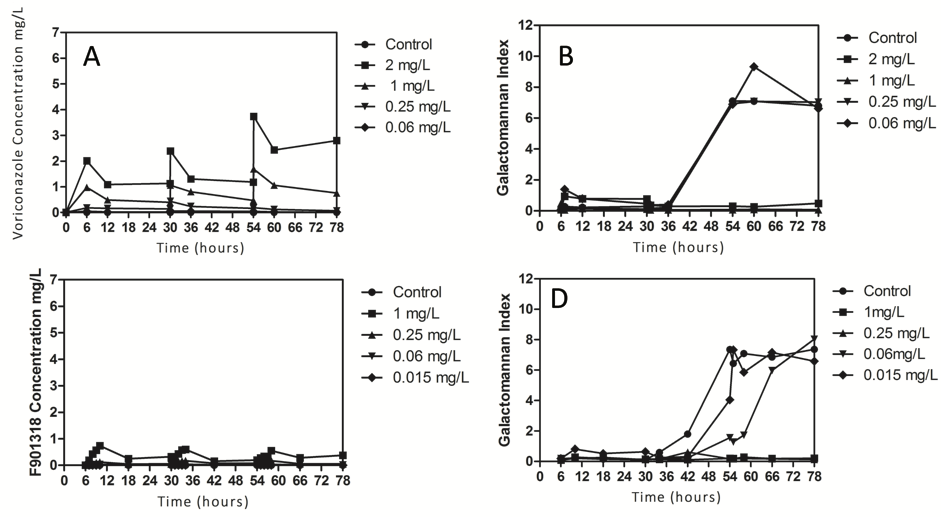
**

**Supplementary Figure 2.** Pharmacokinetics and pharmacodynamics of voriconazole (**A** and **B**) and F901318 (**C** and **D**) in a dynamic model of acute invasive sinusitis caused by *Aspergillus flavus*. The pharmacodynamic readout is the galactomannan index determined using the Platelia kit. The challenge strain was **LEMI1024**. **A***,* Human-like pharmacokinetics of voriconazole; **B***,* pharmacodynamics of voriconazole; **C***,* Human-like pharmacokinetics of F901318; **D***,* pharmacodynamics of F901318.


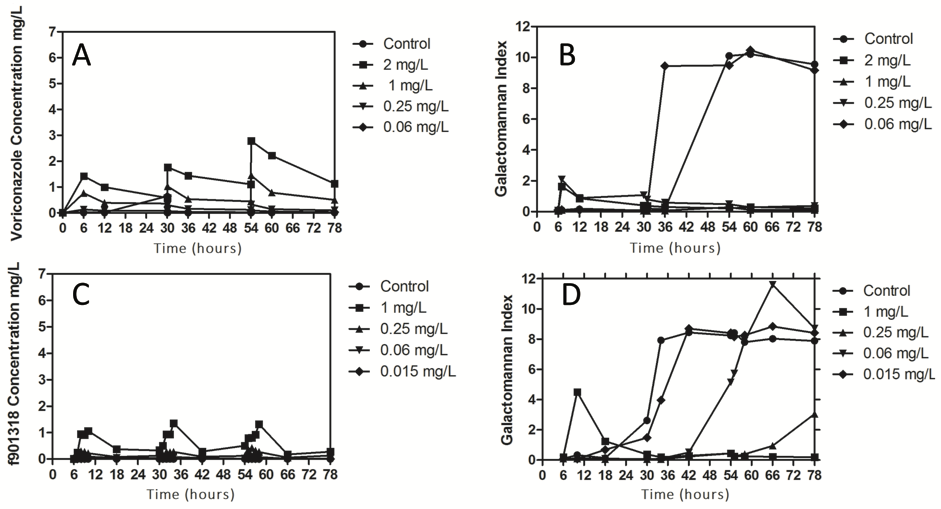


**Supplementary Figure 3.** Pharmacokinetics and pharmacodynamics of voriconazole (**A** and **B**) and F901318 (**C** and **D**) in a dynamic model of acute invasive sinusitis caused by *Aspergillus flavus*. The pharmacodynamic readout is the galactomannan index determined using the Platelia kit. The challenge strain was **LEMI1049**. **A***,* Human-like pharmacokinetics of voriconazole; **B***,* pharmacodynamics of voriconazole; **C***,* Human-like pharmacokinetics of F901318; **D***,* pharmacodynamics of F901318.


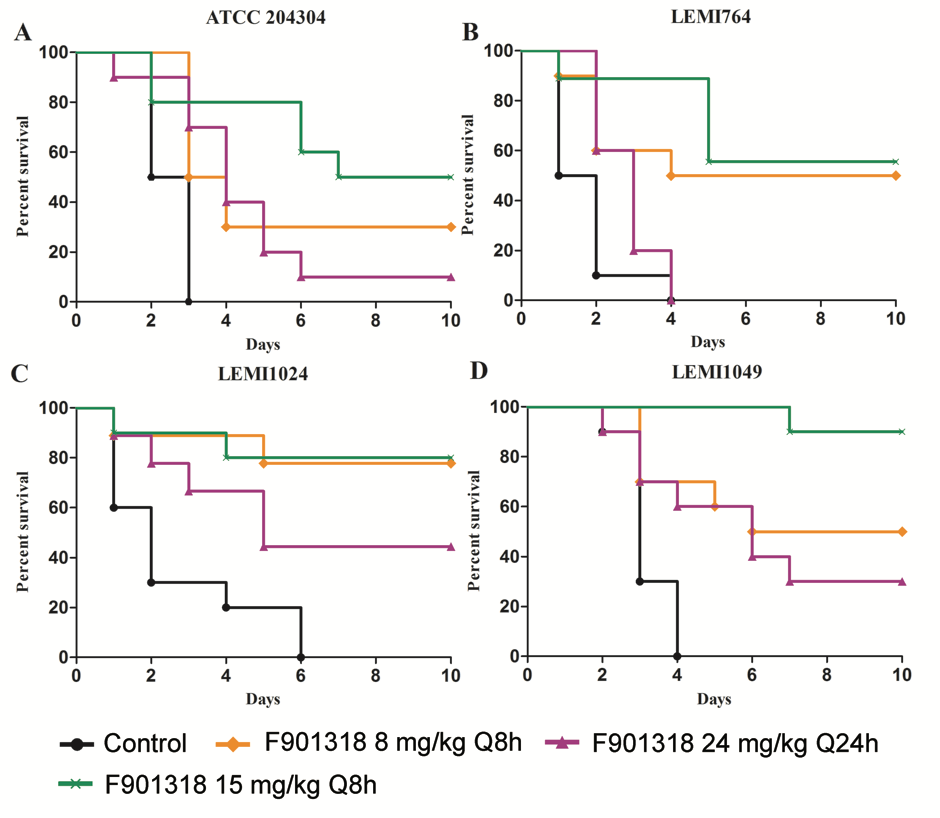


**Supplementary Figure 4.** Survival study in murine model of invasive aspergillosis caused by four different *A. flavus* isolates after treatment with F901318 and posaconazole. **A**, isolate ATCC204304; **B** LEMI764 isolate; **C**, LEMI1024 isolate; **D**, LEMI1049 isolate.
